# Supplementary figures and images for: Individualized diagnosis of rheumatoid arthritis: A rank-based qualitative T cell-related signature
Source: PLoS One. 2025 Jun 26;20(6):e0326027. doi: 10.1371/journal.pone.0326027 (PMC12200850; doi:10.1371/journal.pone.0326027)

**A****Training dataset**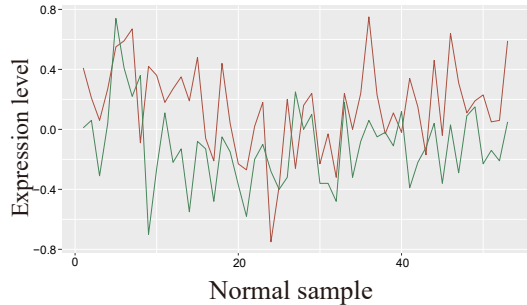**B****Training dataset**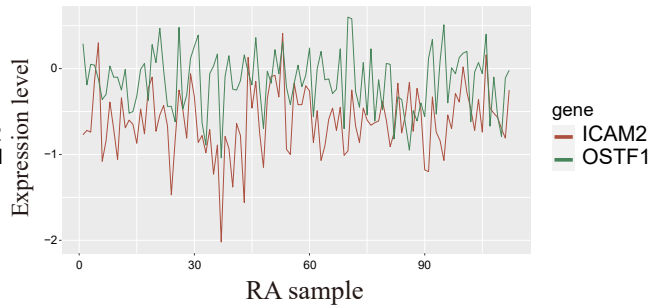

Supplement: S1 Fig — (PDF) [file pone.0326027.s001.pdf]

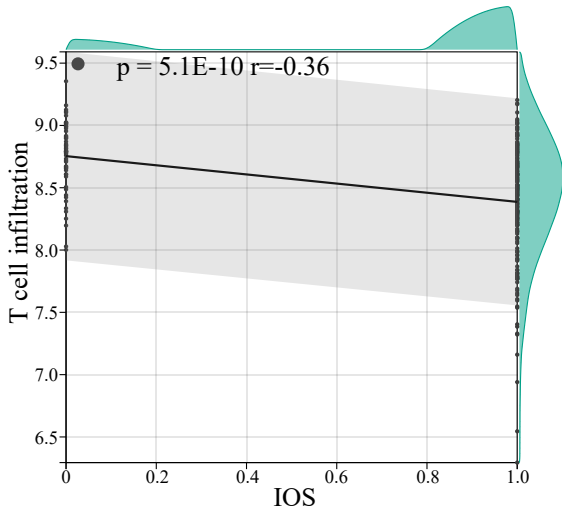

Supplement: S2 Fig — (PDF) [file pone.0326027.s002.pdf]

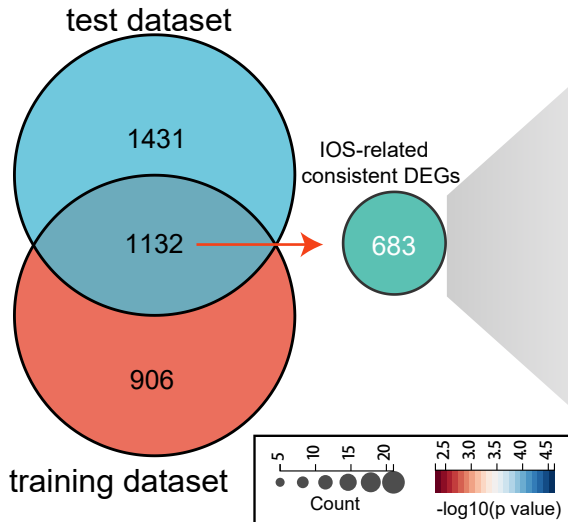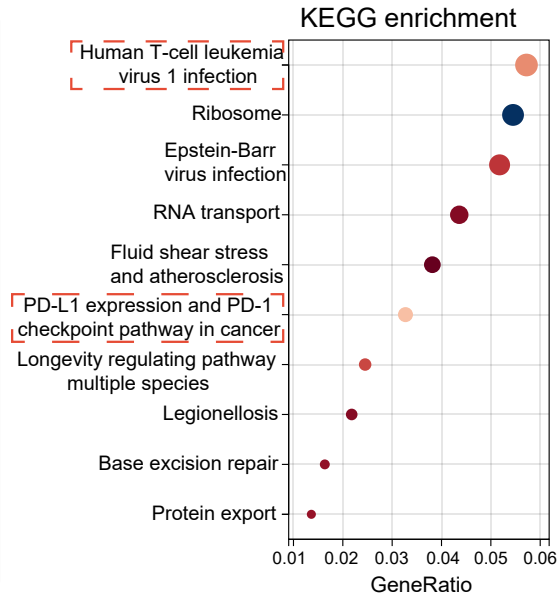

Supplement: S3 Fig — Venn diagram showing the overlap of IOS-related DEGs between the training and test datasets. KEGG pathways significantly enriched with 683 consistent DEGs were determined. p value was detected by hypergeometric distribution model and p < 0.05 was considered significant. (PDF) [file pone.0326027.s003.pdf]

**A****Training dataset**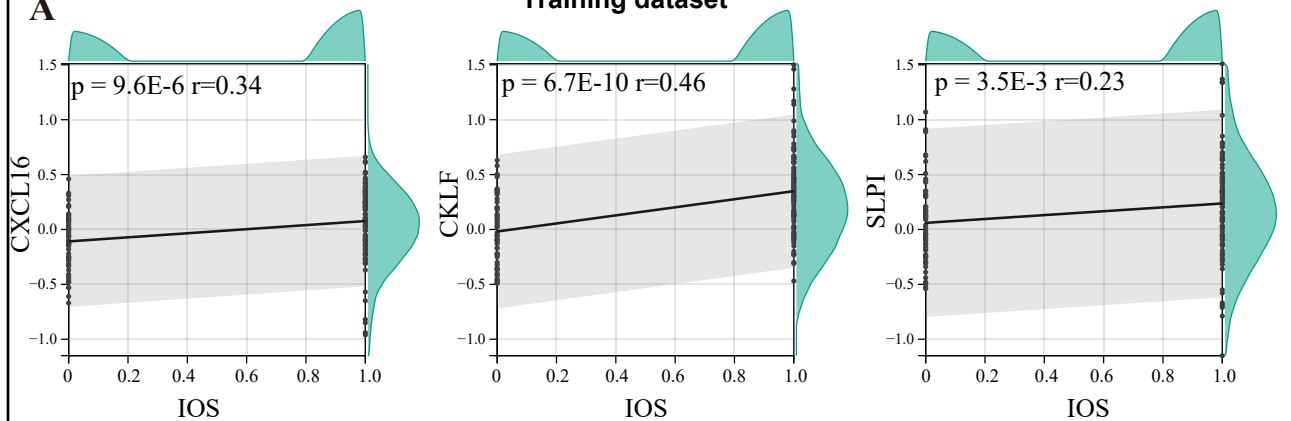**B****Test dataset**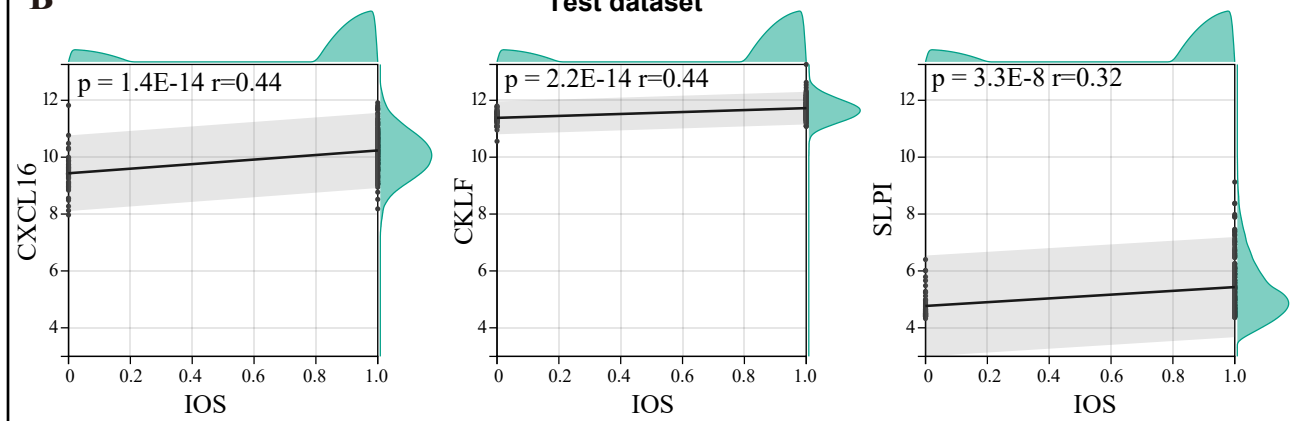

Supplement: S4 Fig — (PDF) [file pone.0326027.s004.pdf]
