# Supplementary material for: Individualized diagnosis of rheumatoid arthritis: A rank-based qualitative T cell-related signature
Source: PLoS One. 2025 Jun 26;20(6):e0326027. doi: 10.1371/journal.pone.0326027 (PMC12200850; doi:10.1371/journal.pone.0326027)
Supplement: S1 Table — (PDF) [file pone.0326027.s005.pdf]

STING1	Interferon response
IFNAR1	Interferon response
IRF7	Interferon response
STAT1	Interferon response
IFIH1	Interferon response
RIGI	Interferon response
TICAM1	Interferon response
IRF3	Interferon response
STAT2	Interferon response
CGAS	Interferon response
JAK1	Interferon response
CDC73	Interferon response
CTR9	Interferon response
LEO1	Interferon response
PAF1	Interferon response
YTHDF2	Interferon response
JAK2	Interferon response
TBK1	Interferon response
TANK	Interferon response
TUFM	Interferon response
TRIM6	Interferon response
TRAF3	Interferon response
CREBBP	Interferon response
MAP1LC3B	Interferon response
NLRC3	Interferon response
AIM2	Interferon response
CCL5	chemokine
CCR5	chemokine
CCL13	chemokine
CCRL2	chemokine
CXCR4	chemokine
CXCL10	chemokine
CCL18	chemokine
CXCL17	chemokine
RIPOR2	chemokine
GNAI1	chemokine
CXCL8	chemokine
TNFAIP6	chemokine
RHOA	chemokine
INPPL1	chemokine
GRK2	chemokine
YWHAB	chemokine
CFL1	chemokine
DOCK8	chemokine
INPP5D	chemokine
SPN	chemokine
MT-RNR1	chemokine
CXCR2	chemokine
CCL2	chemokine
CCR3	chemokine
CCR4	chemokine
CCR2	chemokine
CCR7	chemokine
CCR1	chemokine

CCR6	chemokine
CXCR1	chemokine
CXCR3	chemokine
ACKR3	chemokine
CCL20	chemokine
CCL7	chemokine
CX3CR1	chemokine
ACKR1	chemokine
CCL21	chemokine
CCR8	chemokine
CCR9	chemokine
CXCR5	chemokine
CXCL11	chemokine
CXCL13	chemokine
ACKR2	chemokine
CCL17	chemokine
CCL28	chemokine
CCL3	chemokine
CCL4	chemokine
CXCL2	chemokine
CXCL5	chemokine
CXCL6	chemokine
CXCR6	chemokine
CCL19	chemokine
CCL26	chemokine
CCL8	chemokine
CXCL16	chemokine
CXCL3	chemokine
CXCL9	chemokine
CCL1	chemokine
CCL16	chemokine
CCL22	chemokine
CCL24	chemokine
CCL25	chemokine
XCR1	chemokine
CKLF	chemokine
CXCL14	chemokine
CCL14	chemokine
CCL27	chemokine
CCR10	chemokine
ACKR4	chemokine
CCL15	chemokine
CCL23	chemokine
TAFA2	chemokine
TAFA1	chemokine
TAFA5	chemokine
TAFA4	chemokine
TAFA3	chemokine
CCL3L3	chemokine
CCL3L1	chemokine
CCL4L2	chemokine
CCL4L1	chemokine
CRLF1	Cytokine
SOCS3	Cytokine

CLCF1	Cytokine
MT-RNR1	Cytokine
MYD88	Cytokine
IL1B	Cytokine
TICAM1	Cytokine
MAVS	Cytokine
CGAS	Cytokine
CSF2RB	Cytokine
IL2RG	Cytokine
CISH	Cytokine
SOCS1	Cytokine
SOCS2	Cytokine
SOCS5	Cytokine
SOCS6	Cytokine
CRLF2	Cytokine
CRLF3	Cytokine
GLYR1	Cytokine
CLNK	Cytokine
SOCS4	Cytokine
SOCS7	Cytokine
CYTL1	Cytokine
XCL2	Cytokine
IKBKB	Cytokine
JAK1	Cytokine
STAT1	Cytokine
JAK3	Cytokine
TLR4	Cytokine
IFIH1	Cytokine
TLR5	Cytokine
YWHAQ	Cytokine
IRF8	Cytokine
INPP5D	Cytokine
IRF3	Cytokine
ABCB5	Cytokine
CNTFR	Cytokine
LCP2	Cytokine
NLRP1	Cytokine
STING1	Cytokine
IK	Cytokine
TMEFF2	Cytokine
NLRP6	Cytokine
SH2B2	Cytokine
ZFP36	Cytokine
BUD23	Cytokine
IL17D	Cytokine
NLRP9	Cytokine
NFATC2IP	Cytokine
INAVA	Cytokine
C1QTNF4	Cytokine
SCIMP	Cytokine
TASL	Cytokine
HMGB1	Inflammatory response
TLR4	Inflammatory response
PTGS2	Inflammatory response

PTGS1	Inflammatory response
NLRP9	Inflammatory response
NLRP1	Inflammatory response
NLRP6	Inflammatory response
IKBKG	Inflammatory response
TICAM1	Inflammatory response
NLRP3	Inflammatory response
RIPK1	Inflammatory response
CD163	Inflammatory response
TLR6	Inflammatory response
DHX9	Inflammatory response
TFEB	Inflammatory response
IKBKB	Inflammatory response
MAPK14	Inflammatory response
KEAP1	Inflammatory response
FCGR2B	Inflammatory response
CDK9	Inflammatory response
MAP4K2	Inflammatory response
NR1D1	Inflammatory response
RIPK2	Inflammatory response
GFI1	Inflammatory response
AIM2	Inflammatory response
SLPI	Inflammatory response
BUD23	Inflammatory response
CCL15	Inflammatory response
CCL23	Inflammatory response
