# Supplementary material for: Individualized diagnosis of rheumatoid arthritis: A rank-based qualitative T cell-related signature
Source: PLoS One. 2025 Jun 26;20(6):e0326027. doi: 10.1371/journal.pone.0326027 (PMC12200850; doi:10.1371/journal.pone.0326027)
Supplement: Attachment — Submitted filename: Rebuttal letter.docx [file pone.0326027.s007.docx]

February 27, 2025

Manuscript ID: PONE-D-24-53992

Title: Individualized diagnosis of rheumatoid arthritis: a rank-based qualitative T cell-related signature

Austin W.T. Chiang

Dear Editor at *PLOS ONE*,

Thank you for giving us the opportunity to submit a revised manuscript for publication in the Journal of *PLOS ONE*. We appreciate the time and effort that you and the reviewers dedicated to providing feedback on our manuscript and are grateful for the insightful comments and valuable improvements to our paper.

The following are our point-by-point responses to the reviewers’ comments and critiques have been accommodated fully in various parts of the revised version (shown in BLUE color). We appreciate you and all the reviewers for the generous efforts for our manuscript. Thank you very much.

Sincerely yours,

Yawei Li, Ph.D

School of Biology and Engineering, Guizhou Medical University, Guiyang, Guizhou, China 561113

Tel/fax: 86-155-195-53377

Email: liyawei@gmc.edu.cn

**Journal Requirements:**

1. When submitting your revision, we need you to address these additional requirements.

Please ensure that your manuscript meets PLOS ONE's style requirements, including those for file naming. The PLOS ONE style templates can be found at 
https://journals.plos.org/plosone/s/file?id=wjVg/PLOSOne_formatting_sample_main_body.pdf and

https://journals.plos.org/plosone/s/file?id=ba62/PLOSOne_formatting_sample_title_authors_affiliations.pdf

**Reply 1: Thanks. Done as the requested.**

2. We noticed you have some minor occurrence of overlapping text with the following previous publication(s), which needs to be addressed:

https://wjso.biomedcentral.com/articles/10.1186/s12957-024-03314-8

In your revision ensure you cite all your sources (including your own works), and quote or rephrase any duplicated text outside the methods section. Further consideration is dependent on these concerns being addressed.

**Reply 2: Thanks.** **The description that may involve overlap has been modified. Correction are as follows.**

**In the revised Materials and methods in lines 83-88: “The same preprocessing was performed for these datasets, the gene expression profiles (series.txt) were directly used after the process of probe re-annotation without any normalization. The probe set IDs were mapped to Entrez gene IDs with the corresponding platform annotation file for all gene expression profile data. If multiple probe sets were mapped to the same gene, the expression value of the gene was calculated by its average value. Besides, if a probe mapped multiple Entrez gene IDs or did not map any Entrez gene ID, the probe was deleted.”** **🡪 “The identical preprocessing was executed for these datasets, with the gene expression profiles (series.txt) utilized directly following the probe re-annotation process, devoid of any normalization. The probe set IDs were correlated with Entrez gene IDs using the relevant platform annotation file for all gene expression profile data. If numerous probe sets corresponded to the same gene, the expression value was determined by calculating its average value. Furthermore, probes that mapped multiple Entrez gene IDs or failed to map any Entrez gene ID were eliminated.”;**

**In the revised Results in lines 157-158: “With the majority voting rule, 97 of 112 RA samples and 46 of 53 healthy samples in the training dataset were correctly classified” 🡪“97 out of 112 RA samples and 46 out of 53 healthy samples were accurately categorized**.**”;**

**In the revised Results in lines 175-178: “It is known that the** **occurrence and development of RA are related to immune microenvironments. To determine the relation between RA and immune microenvironments, we composed a heatmap to visualize the abundance of 28 infiltrating immune cell populations of 280 samples from the test datasets using the ssGSEA method.” 🡪 “****The occurrence and development of RA are associated with immunological microenvironments. Utilizing the ssGSEA approach, the abundance of 28 infiltrating immune cell types was illustrated using 280 samples from the testing datasets to investigate the correlation between RA and microenvironments.”;**

**In the revised Results in lines 178-179: “Results showed that RA had a higher abundance of immune cell infiltration (e.g., activated dendritic cell, activated CD8 T cell, and Type 1 T helper cell, Figure 3A).” 🡪 “****The results indicated that RA exhibited a greater abundance of immune cell infiltration, including activated dendritic cells, activated CD8 T cells, and type 1 T helper cells (Fig 3A).****”;**

**In the revised Results in lines 182-183: “Here, we integrated seven popular algorithms to estimate the infiltrating immune cell fraction in RA samples.” 🡪 “****Further, seven widely used algorithms were employed to calculate the infiltrating immune cell fraction in RA samples.”;**

**In the revised Results in line 183: “Compared with healthy samples” 🡪 “In comparison to healthy samples”; “showed” 🡪 “exhibited”; “higher fraction”** **🡪“elevated proportion”;**

**In line 184: “On the contrary”🡪“In contrast”;**

**In lines 185-186: “showed a lower fraction of” 🡪 “****exhibited a reduced proportion of”; “,** **in over two algorithms” 🡪 “across more than two algorithms”;**

**In the revised Discussion in lines 310-312: “IOS should be verified in a prospective clinical investigation, which might reduce unnecessary expenses for the early detection of RA.” 🡪 “****IOS requires verification in a prospective clinical investigation to potentially minimize unnecessary costs associated with the early detection of RA.”**

3. We note that the grant information you provided in the ‘Funding Information’ and ‘Financial Disclosure’ sections do not match.

When you resubmit, please ensure that you provide the correct grant numbers for the awards you received for your study in the ‘Funding Information’ section.

**Reply 3: Thanks. Done as the requested. We provided the correct funding information in the Funding section in lines 333-335: “This research was supported by a grant from** **the Basic Research Program of Guizhou Science and Technology Department (ZK[2024]156) and the High-level Talents Startup Fund of Guizhou Medical University (J[2022]042).”**

4. Please note that your Data Availability Statement is currently missing the direct link to access each database. If your manuscript is accepted for publication, you will be asked to provide these details on a very short timeline. We therefore suggest that you provide this information now, though we will not hold up the peer review process if you are unable.

**Reply 2: Thanks.** **We have added the direct link of data used in the present study. In the revised Data availability: “The datasets analyzed during the present study are available from the Gene Expression Omnibus (https://www.ncbi.nlm.nih.gov/geo/query/acc.cgi?acc=GSE17755**; **https://www.ncbi.nlm.nih.gov/geo/query/acc.cgi?acc=GSE97475;https://www.ncbi.nlm.nih.gov/geo/query/acc.cgi?acc=GSE97810)”**

5. Please include captions for your Supporting Information files at the end of your manuscript, and update any in-text citations to match accordingly. Please see our Supporting Information guidelines for more information: http://journals.plos.org/plosone/s/supporting-information.

**Reply 5: Thanks. Done as the requested.**

**Review Comments to the Author**

Reviewer #1:

Introduction

1. Why do the serum biomarkers currently used to detect RA in clinical practice lack sufficient accuracy? Can these biomarkers be used to explain the heterogeneous features of RA patients? If so, how can their accuracy be improved?

**Reply 1: Thanks for your comments. Rheumatoid factor (RF) and Anti-cyclic citrullinated** **peptide antibody (ACPA) are currently used for RA diagnosis in the clinic. However, they also can be found in other connective tissue diseases, some chronic infectious diseases, or systemic lupus erythematosus, leads to a poor accuracy [1, 2]. Besides,** **since the diagnosis of RA mainly relies on the expression measurement of serum biomarkers, batch effects may also affect the test results [3].** **They do explain, to some extent, the heterogeneity of RA. For example, patients with higher titers of RF and anti-CCP antibodies tend to have more severe joint destruction and may progress more quickly [4, 5]. This suggests that there is heterogeneity in the severity of the disease among patients with different antibody levels. Therefore, a potential method to improve their accuracy is to combine with indicators related to the degree of inflammatory activity of the disease such as C-reactive protein and erythrocyte sedimentation rate for RA diagnosis.**

2. How can signatures developed based on within-sample relative expression orderings (REOs) of gene pairs be used to explain the heterogeneous features of RA patients?

**Reply 2: Thanks for your comments. The gene pair signature is not used to explain the heterogeneous features of RA patients but to overcome the batch effects across heterogeneous patient cohorts, which is a major obstacle to RA early detection.**

**We have re-described in Introduction in lines 50-53:** “**In contrast, signatures developed based on within-sample relative expression orderings (REOs) of gene pairs are resistant to batch effects across heterogeneous patient cohorts or normalization effects by different technical sources and can be applied to individual samples [6-8].**”

3. Regarding the role of T cells, can the author explain more clearly why T cells are necessary for the onset and progression of RA?

**Reply 3: Thanks. The immunomodulatory imbalance induced by T cells also promotes the occurrence and progression of RA. For example, in healthy humans, Th1 and Th2 cells are in balance to maintain immunological homeostasis. However, Th1 cells are predominant in RA patients. The increased secretion of cytokines such as interferon-γ by Th1 cells can promote the activation of immune cells such as macrophages, enhance cellular immune response, lead to aggravated inflammatory response, and promote the occurrence and development of RA [9]. Besides, the balance between Th17 and Treg cells is also essential for maintaining immune tolerance and immune homeostasis. In RA, Th17 cell function is enhanced and Treg cell function is relatively insufficient. This imbalance prevents the body from properly regulating the immune response against its own joint tissues, which results in RA [10].**

Materials and Methods

1. Concerning the mapping of probe set IDs, has the author considered about calculating a gene’s expression value by using its maximum value among the corresponding probe sets? If so, what were the effects?

**Reply 1: Thanks, in probe re-annotation, individual extreme values may appear due to the experimental errors, if the maximum value is used, these outliers may have a greater impact on the results, leading to an incorrect estimate of the gene expression level. The average value can reduce the interference of outliers to a certain extent by averaging all probes, and more stably reflect the true expression of genes. Therefore, we used the average value rather than the maximum value.**

2. What are the advantages of the REO method compared to traditional correlation-based methods?

**Reply 2: Thanks, REO method, unlike traditional correlation-based methods, has been reported that: (****i) is resistant to experimental and technical variations; (ii) is invariant to monotonic data normalization; (iii) can be applied to individual samples without a complex scoring process [3, 11].**

**3. Can the REO method be applied to certain aspects of immunological feature analysis and network analysis?**

**Reply 3: Thanks. At present, our laboratory uses the REO method, considering its advantages, mainly in three aspects: (i) the discovery of disease biomarkers [7, 8]; (ii) the identification of individual pathways [12]; (iii) the identification of individualized differentially expressed genes [13]. (ii) and (iii) may be involved in immunological feature analysis and network analysis.**

Results

1. Why does only one gene pair, ICAM2-OSTF1, overlap between TRGPs and the reversal gene pairs? Could other results be obtained if the methods were optimized?

**Reply 1: This result mainly depends on the data we used in the present study, especially since only T cell-related gene expression profiles were involved. In this context, even if alternative gene pairs are identified through method optimization, their diagnostic performance is inferior to that of the ICAM2 - OSTF1.**

2. The sentence "the REO patterns of IOS are stably" should be corrected to "the REO patterns of IOS are stable."

**Reply 2:** **Thanks. Done as suggested.**

3. Could the author provide more details on the correlation between ICAM2-OSTF1 and T-cell infiltration?

**Reply 3: Thanks. Done as suggested. We have added the related result in the revised Result in lines 190-191: “****Besides, ICAM2-OSTF1 was found negative correlation with T cell infiltration (r=-0.36, spearman’s rank correlation, S2 Fig).”**

**S2 Fig. Relationship between ISO and T cell infiltration calculated by MCPCOUNTER.**

4. Could the author provide more details on the correlation between ICAM2-OSTF1 and T-cell immune pathways?

**Reply 4: Thanks. We could not directly evaluate the correlation because the individual pathway scores cannot be quantitative. However, using the wilcoxon rank-sum test with FDR < 0.01 control, 683 IOS-related DEGs were identified between groups classified by IOS. And these DEGs participated T cell immune-related pathways such as “Human T-cell leukemia virus 1 infection” and “PD-L1 expression and PD-1 checkpoint pathway in cancer” [14, 15]. The related descriptions are in lines 218-221: “Similarly, using the wilcoxon rank-sum test with FDR < 0.01 control, 683 IOS-related DEGs were identified between groups classified by IOS. And these DEGs participated T cell immune-related pathways such as “Human T-cell leukemia virus 1 infection” and “PD-L1 expression and PD-1 checkpoint pathway in cancer” (*p*<0.05, S3 Fig)”**

**S3 Fig.Venn diagram showing the overlap of IOS-related DEGs between the training and test datasets. KEGG pathways significantly enriched with 683 consistent DEGs were determined. *p* value was detected by hypergeometric distribution model and *p* < 0.05 was considered significant.**

Discussion

1. How can the REO method outperform other approaches in explaining the heterogeneous features of RA patients?

**Reply 1: Thanks. Please see Reply 2 in the Introduction.**
2. Why are the three key inflammation-related genes (CXCL16, CKLF, and SLPI) discussed?

What is their relationship with ICAM2-OSTF1?

**Reply 2: Thanks.** ***CXCL16*, *CKLF*, and *SLPI* are with significantly differentially expressed** **inflammation-related genes between RA and normal sample with large degrees in the network (Fig 5), which are more possibility to have potential regulatory effects on RA occurrence or development. According to the reviewer’s suggestion, we evaluated their relationship. We have added relation descriptions in the revised Results in lines 255-257: “****The relationship between IOS and these three genes was also evaluated. In addition to *SLPI*, a moderate positive correlation between IOS and the expression levels of other genes was achieved in training and test datasets (r>0.3, spearman’s rank correlation, S4 Fig).” and in the revised Discussion in lines 302-303: “We also found that individual IOS status was positively correlated with these three key genes.”**

**S4 Fig. Relationship between ISO and the expression of *CXCL16*, *CKLF*, and *SLPI* in (A) training dataset and (B) test dataset, respectively.**

3. Could the author describe the biological significance of the ICAM2-OSTF1-related network, and how it can be validated experimentally?

**Reply 3:** **Thanks. In the present study, this network was constructed based on the correlation matrix of expression, thus we cannot obtain the direct regulatory relationship between these differentially expressed genes. However, as described in Reply 2, we found four genes (*CXCL16*, *CKLF*, *SLPI* and *ICAM2*) achieved a large degree in the network, which were considered as potential regulators of RA occurrence and development. Currently, the conditions for functional verification of hub genes are not yet available, which requires animal models, cell models, knockout and other experimental technologies. We have discussed the related description in the limitation of the study in lines 306-308: “****Besides, when focusing on the construction of the network, we were unable to verify the function of hub genes through biological experiments. However, enrichment analysis was used to investigate the pathways that these genes may affect.”**

**Reviewer #2:**

1. In Materials and Methods section, in terms of the validation dataset, the normal sample and RA sample are from two dataset. There will be potential bias on these dataset design and batch effect. How do you address these problems?
**Reply 1: Thanks for your comments. The REO method we used in this study is resistant to batch effects or normalization effects by different technical sources and can be applied to individual samples. The related description is in the Introduction in lines 51-54.**

2. While the validation datasets are independent, they originate from similar sources (peripheral blood mononuclear cells). If possible, including other type of dataset could further validate your model’s robustness.

**Reply 2: Thanks for your suggestion. However, processing methods are highly variable if sample sources are different, leading to distinct expression patterns [16]. Therefore, our signature did not apply to other types of samples except for peripheral blood samples.**

Minor issues
1. Line 150, “97 of 111 RA samples”, is total should be 112?

**Reply 1: We would like to express our sincere appreciation for your help to our manuscript. In the revised Results in line 156: “97 of 111 RA samples” 🡪 “97 out of 112 RA samples”**

2. For the figures, it will be better to save them in higher resolution files.
**Reply 2: Thanks, done as suggested. The resolution of all figures changed from 400 ppi to 600 ppi.**

In conclusion, the paper introduces a novel diagnostic framework with potential clinical impact. Addressing the comments above would enhance its translational application in RA diagnosis.

**Reviewer #3:**

The manuscript is logically well-structured and presents its finding effectively. However, it contains many typos and grammatical errors, some of which have been identified. To improve the overall quality and readability, the language should be carefully revised and polished, preferably by a native English speaker.
**Reply: We would like to express our sincere appreciation for your help to our manuscript. We have carefully grammatical errors in the whole manuscript and corrected these errors in the revised manuscript. Correction are as follows.**

**In the revised Abstract in line 18: “disease with individual persistent synovitis” 🡪 “disease with persistent synovitis”;**

**In line 24: “validation dataset that contain a total” 🡪 “validation dataset that** **contained a total”**

**In the revised Introduction in line 43: “in clinical” 🡪“in clinical settings”;**

**In line 45: “signature for the detection RA” 🡪“signature for the detection of RA”;**

**In line 56: “interferon response” 🡪 “interferon responses”; “Regulatory” 🡪 “regulatory”;**

**In line 57: “immunity balanced”🡪“immunity balance”; “the** **dysfunction of which contributes to RA pathogenesis” 🡪“****whose dysfunction contributes to RA pathogenesis”;**

**In the revised Materials and methods in line 72: “Data collecting” 🡪“Data collection”;**

**In line 77: “which was including” 🡪“which includes”;**

**In line 92: “20 Regulatory T cell-related genes” 🡪“20 regulatory T cell-related genes”;**

**In line 96: “, respectively,” 🡪“, respectively.”;**

**In line 97: “defined as that whose REO” 🡪“defined as those whose REO”;**

**In the revised Results in line 143: “samples, but” 🡪“samples but”;**

**In line 146: “****pairs, was considered” 🡪“pairs and was considered”;**

**In line 156: “The AUC of IOS” 🡪“the AUC of IOS”;**

**In line 157: “The accuracy of IOS were” 🡪“The accuracy of IOS was”;**

**In line 160: “93.94” 🡪“93.94%”;**

**In line 165: “**I**OS are stably” 🡪“IOS are stable”;**

**In line 185: “naiver” 🡪“naïve”;**

**In line 226: “over-expressed” 🡪“overexpressed”;**

**In line 227: “thus might leading to” 🡪“thus possibly leading to”;**

**In line 254: “genes driving” 🡪“genes are driving”;**

**In the revised Discussion**

**In line 266:** delete **“ion channel genes”**

**In the revised Figure legend**

**In line 152: “Workflow of identification” 🡪“Workflow for the identification”;**

**In line 198: “into a z-score” 🡪“with a z-score”;**

**In lines 198-199: “Fraction of immune cells in 280 samples calculated” 🡪“The fraction of immune cells in 280 samples is calculated”;**

**In line 201:** “**fraction in RA**” 🡪“**fractions in RA”;**

1. In line 58, the phrase “immunity balanced” is grammatically incorrect. It should be revised to “immune balance”. Please consider rephrasing as “whose dysfunction contributes…” instead of “the dysfunction of which contributes…” for improved clarity.

**Reply 1: Thanks. Done as suggested.**

2. In line 78, “which was including” is grammatically incorrect. Please replace it with “Which includes” for better grammar and readability.

**Reply 2: Thanks. Done as suggested. Please see Reply above.**

3. The quality of the figures needs improvement. Some of them are not readable. Please ensure they are clear and legible.

**Reply 3: Thanks. Done as suggested. The resolution of all figures changed from 400 ppi to 600 ppi.**

4. In the sentence spanning lines 121 to 123, there are multiple grammar error, please rephrase it for clarity.

**Reply 4: Thanks. Done as suggested. The original sentence was changed to “****The one-sided Wilcoxon rank-sum test was employed to compare the aforementioned immunity features between normal and rheumatoid arthritis samples. Spearman rank correlation was used to assess the relationships between the expression levels of two signature genes and the immune infiltration of 28 immune cells.”**

5. Delete first “and” in line 122.

**Reply 5: Thanks. Done as suggested.**

6. Figure 2F is not mentioned in the manuscript. Please ensure that all figures are properly described and mentioned with in the text.

**Reply 6: Thanks. Done as suggested. In line 157-158: “while in the RA samples the fold change took values ranging from 0.85 to 1.06 with the median of 0.96 (Figure 2F).”**

7. The X-axis label in Figure 2F is “Tumor sample”, but I did not see any tumor samples in the manuscript. Should this be corrected to “RA samples”?

**Reply 7 Thanks. Done as suggested.**

8. In line 172, replace “naiver” change to “naïve”.

**Reply 8: Thanks. Done as suggested.**

9. In Figure 3B, monocytes and activated NK cells appear to increase in RA samples, but the author did not mention them in results section. Similarly, decreased fractions like activated CD4 memory T cells and gamma delta T cells were not discussed. These findings should be included for completeness.

**Reply 9: Thanks. We are sorry for the complete description. We have re-described this sentence in the revised Results in line 182-184: “RA samples exhibited a significantly elevated proportion of monocyte, myeloid dendritic cell, activated myeloid dendritic cell, neutrophil and activated NK cell, in over two algorithms (*p* < 0.05, Wilcoxon rank-sum test, Fig 3B).” and in the revised Discussion in line 275-279: “The RA samples exhibited an increased abundance of five immune cell types, namely monocytes, myeloid dendritic cells, activated myeloid dendritic cells, neutrophils, and activated NK cells, while three immune cell types, specifically activated CD4+ memory T cells, naïve CD4+ T cells, and gamma delta T cells, showed a decreased presence.”**

10. Figure 3, 4 and 5 are not readable in its current form. Enhance its quality for clarity and ensure all labels and data are visible.

**Reply 10: Thanks. Done as suggested. We have increased the font size in figures to ensure it is larger than 6pt.**

11. The term "800 consistent DEGs" needs definition. Do these include only upregulated genes, or do they encompass both upregulated and downregulated genes?

**Reply 9: Thanks. We are sorry for the confused description. We have re-defined it in the revised Materials and methods in lines 112-114: “Further, the overlaps with consistent dysregulation between these two gene lists were considered consistent DEGs.”**

12. In Figure 4B, please consider using the adjusted p-value on the X-axis instead of the number of genes to enhance interpretability.

**Reply 7 Thanks. Done as suggested.**

**Fig 4. Analysis of transcriptome differences between RA and normal samples.**

13. In Figure 4, several immune-related pathways (e.g., neutrophil activation and T-cell activation) are mentioned, but the manuscript lacks further discussion on how these pathways influence the onset and progression of RA. Please either conduct a more detailed analysis of these pathways or reference existing literature discussing their roles in RA.

**Reply 14: Thanks. We have added the related description in the revised Discussion in lines 281-291: “****For instance, neutrophils are frequently detected in the synovial fluid in RA. Their presence in the synovial fluid is implicated in the induction of local inflammation, tissue damage, and erosion [17, 18]. The “neutrophil activation” pathway could unleash proteolytic enzymes. These enzymes degrade extracellular matrix components in joints, harming cartilage and bone, and thus playing a significant role in the progression of RA [19, 20]. “T cell activation” pathway can regulate the balance of different T-cell subsets such as helper T cells and regulatory T cells [21]. In patients with RA, there is usually an increase in type 17 T helper cells and impaired function of regulatory T cells [22]. Cytokines secreted by type 17 T helper cells cells, such as interleukin - 17, can promote the inflammatory response. However, the abnormal function of regulatory T cells fails to effectively suppress the immune response, leading to immune imbalance and exacerbating the condition of RA [23, 24].”**

14. In lines 190-191, clarity how the authors localized inflammatory-related genes. Was this derived from KEGG analysis? I think your input genes for KEGG analysis are dysregulated gene already. Please clarify.

**Reply 14: Thanks. We are sorry for the confused description. We have re-described how to get the** **inflammatory-related gene list in the revised Materials and methods in lines 114-117: “****Inflammatory-related gene lists were obtained from the GeneCards database (https://www.genecards.org/) utilizing four keywords: “Interferon response,” “chemokine,” “Cytokine,” and “Inflammatory response,” under the categories “Protein Coding” and “Proteins” (S1 Table).” and in the revised Results in lines 222-224: “Additionally,** **when localizing** **inflammatory-related genes such as cytokine, inflammatory response, interferon response and chemokine, we found that some genes were also dysregulated in RA (Fig 4C-D)”. Here, we focus on the differentially expressed inflammatory-related genes, which have potential biological significance for the occurrence and development of RA.**
15. From lines 193 to 196, the author mentioned two RA related Genes CKLF and JAK3, then the author state that these genes are associated with dysfunction of the immune microenvironment in RA. However, there is no evidence presented on how these genes affect the immune microenvironment. Please provide references to relevant studies and avoid over-concluding.

**Reply 14: Thanks. We are sorry for the inaccurate description. We have re-described it in the revised Results in line 227: “These genes were associated with immune or inflammatory responses [25, 26]”**

16. In line 218, the mention of ion channel genes is unrelated and confusing, as these genes are not discussed elsewhere in the manuscript. Clarify or remove this statement.

**Reply 16: Thanks. We have removed this statement.**

**Reference**

1.	Sutton B, Corper A, Bonagura V, Taussig M. The structure and origin of rheumatoid factors. Immunology today. 2000;21(4):177-83.

2.	Zhao Y, Li J, Li XX, Li C, Li L, Li ZG. What can we learn from the presence of anti-cyclic citrullinated peptide antibodies in systemic lupus erythematosus? Joint bone spine. 2009;76(5):501-7.

3.	Guan Q, Yan H, Chen Y, Zheng B, Cai H, He J, et al. Quantitative or qualitative transcriptional diagnostic signatures? A case study for colorectal cancer. BMC genomics. 2018;19(1):99.

4.	Oliveira V, Reis A, Brenol CV, Pereira IA, Bonfiglioli KR, Pereira LR, et al. High-Titer Rheumatoid Factor is Associated with Worse Clinical Outcomes and Higher Needs for Advanced Therapies in Rheumatoid Arthritis Under Real-Life Conditions. Rheumatology and therapy. 2025;12(1):123-36.

5.	de Brito Rocha S, Baldo DC, Andrade LEC. Clinical and pathophysiologic relevance of autoantibodies in rheumatoid arthritis. Advances in rheumatology (London, England). 2019;59(1):2.

6.	Li Y, Zhao Z, Ai L, Wang Y, Liu K, Chen B, et al. Discovering a qualitative transcriptional signature of homologous recombination defectiveness for prostate cancer. iScience. 2021;24(10):103135.

7.	Liu K, Geng Y, Wang L, Xu H, Zou M, Li Y, et al. Systematic exploration of the underlying mechanism of gemcitabine resistance in pancreatic adenocarcinoma. Molecular oncology. 2022;16(16):3034-51.

8.	Chen T, Yu T, Zhuang S, Geng Y, Xue J, Wang J, et al. Upregulation of CXCL1 and LY9 contributes to BRCAness in ovarian cancer and mediates response to PARPi and immune checkpoint blockade. British journal of cancer. 2022;127(5):916-26.

9.	Schulze-Koops H, Kalden JR. The balance of Th1/Th2 cytokines in rheumatoid arthritis. Best practice & research Clinical rheumatology. 2001;15(5):677-91.

10.	Jiang Q, Yang G, Liu Q, Wang S, Cui D. Function and Role of Regulatory T Cells in Rheumatoid Arthritis. Frontiers in immunology. 2021;12:626193.

11.	Qi L, Chen L, Li Y, Qin Y, Pan R, Zhao W, et al. Critical limitations of prognostic signatures based on risk scores summarized from gene expression levels: a case study for resected stage I non-small-cell lung cancer. Briefings in bioinformatics. 2016;17(2):233-42.

12.	Zhang J, Xiao H, Song K, Li K, Yuan H, Yuan R, et al. IndGOterm: a qualitative method for the identification of individually dysregulated GO terms in cancer. Briefings in bioinformatics. 2022;23(2).

13.	Cai H, Chen L, Yang S, Jiang R, Guo Y, He M, et al. Personalized differential expression analysis in triple-negative breast cancer. Briefings in functional genomics. 2024;23(4):495-506.

14.	Olière S, Douville R, Sze A, Belgnaoui SM, Hiscott J. Modulation of innate immune responses during human T-cell leukemia virus (HTLV-1) pathogenesis. Cytokine & growth factor reviews. 2011;22(4):197-210.

15.	Lin X, Kang K, Chen P, Zeng Z, Li G, Xiong W, et al. Regulatory mechanisms of PD-1/PD-L1 in cancers. Molecular cancer. 2024;23(1):108.

16.	Gautam A, Donohue D, Hoke A, Miller SA, Srinivasan S, Sowe B, et al. Investigating gene expression profiles of whole blood and peripheral blood mononuclear cells using multiple collection and processing methods. PloS one. 2019;14(12):e0225137.

17.	Wright HL, Moots RJ, Edwards SW. The multifactorial role of neutrophils in rheumatoid arthritis. Nature reviews Rheumatology. 2014;10(10):593-601.

18.	Mayadas TN, Cullere X, Lowell CA. The multifaceted functions of neutrophils. Annual review of pathology. 2014;9:181-218.

19.	Zhang L, Yuan Y, Xu Q, Jiang Z, Chu CQ. Contribution of neutrophils in the pathogenesis of rheumatoid arthritis. Journal of biomedical research. 2019;34(2):86-93.

20.	Khandpur R, Carmona-Rivera C, Vivekanandan-Giri A, Gizinski A, Yalavarthi S, Knight JS, et al. NETs are a source of citrullinated autoantigens and stimulate inflammatory responses in rheumatoid arthritis. Science translational medicine. 2013;5(178):178ra40.

21.	Li MO, Rudensky AY. T cell receptor signalling in the control of regulatory T cell differentiation and function. Nature reviews Immunology. 2016;16(4):220-33.

22.	Wang T, Rui J, Shan W, Xue F, Feng D, Dong L, et al. Imbalance of Th17, Treg, and helper innate lymphoid cell in the peripheral blood of patients with rheumatoid arthritis. Clinical rheumatology. 2022;41(12):3837-49.

23.	Cope AP, Schulze-Koops H, Aringer M. The central role of T cells in rheumatoid arthritis. Clinical and experimental rheumatology. 2007;25(5 Suppl 46):S4-11.

24.	Weyand CM, Bryl E, Goronzy JJ. The role of T cells in rheumatoid arthritis. Archivum immunologiae et therapiae experimentalis. 2000;48(5):429-35.

25.	Li Y, Yu H, Feng J. Role of chemokine-like factor 1 as an inflammatory marker in diseases. Frontiers in immunology. 2023;14:1085154.

26.	Lü L, Yakoumatos L, Ren J, Duan X, Zhou H, Gu Z, et al. JAK3 restrains inflammatory responses and protects against periodontal disease through Wnt3a signaling. FASEB journal : official publication of the Federation of American Societies for Experimental Biology. 2020;34(7):9120-40.
