# Supplementary material for: Individualized diagnosis of rheumatoid arthritis: A rank-based qualitative T cell-related signature
Source: PLoS One. 2025 Jun 26;20(6):e0326027. doi: 10.1371/journal.pone.0326027 (PMC12200850; doi:10.1371/journal.pone.0326027)
Supplement: Attachment — Submitted filename: Rebuttal letter-2.docx [file pone.0326027.s008.docx]

May 11, 2025

Manuscript ID: PONE-D-24-53992

Title: Individualized diagnosis of rheumatoid arthritis: a rank-based qualitative T cell-related signature

Wan-Tien Chiang

Dear Academic Editor at *PLOS ONE*,

Thank you for giving us the opportunity to submit a revised manuscript for publication in the Journal of *PLOS ONE*. We appreciate the time and effort that you and the reviewers dedicated to providing feedback on our manuscript and are grateful for the insightful comments and valuable improvements to our paper.

All the reviewers' comments have been addressed, **except for two remaining concerns from Reviewer #3**. The following are our point-by-point responses to the reviewer #3 comments and critiques have been accommodated fully in various parts of the revised version (shown in BLUE color). We appreciate you and all the reviewers for the generous efforts for our manuscript. Thank you very much.

Sincerely yours,

Yawei Li, Ph.D

School of Biology and Engineering, Guizhou Medical University, Guiyang, Guizhou, China 561113

Tel/fax: 86-155-195-53377

Email: liyawei@gmc.edu.cn

**Journal Requirements:**

Please review your reference list to ensure that it is complete and correct. If you have cited papers that have been retracted, please include the rationale for doing so in the manuscript text, or remove these references and replace them with relevant current references. Any changes to the reference list should be mentioned in the rebuttal letter that accompanies your revised manuscript. If you need to cite a retracted article, indicate the article’s retracted status in the References list and also include a citation and full reference for the retraction notice.

**Reply: Thanks. All the references are cited correctly, and no article has been retracted.**

**Review Comments to the Author**

Reviewer #1: The corrections fully support a value of the method----within-sample relative expression orderings (REOs) of gene pairs for diagnosis of rheumatoid arthritis. All parts in this manuscript which should be corretced have been corrected.

**Reply: We thank the reviewer for the agreement with the main findings of our work.**

Reviewer #2: (No Response)

Reviewer #3: Thank you for your prompt response and revision. Most of my concerns have been addressed, and the manuscript has been significantly improved. However, I still have two remaining concerns:

1. In line 58, the phrase "immunity balanced" is grammatically incorrect and has not been corrected. Please replace it with "immune balance."

**Reply: We would like to express our sincere appreciation for your help to our manuscript. "immunity balanced" have been replaced with "immune balance."**

2. In response to comment 12, the authors have provided a high-resolution figure for Figure 4. However, I did not notice any changes to Figures 3 and 5, as mentioned in comment 10.
**Reply: To make the Figures more legible, we have increased the font size of 3B-C and 5B-E. The resolution of figures 3 and 5 has also changed from 400 ppi to 600 ppi. It is possible that the PDF document generation system causes the figures to appear unclear, but this is clearly stated in the doc file. I have listed the revised figures 3 and 5 below.**

**Fig 3. Immune infiltration analysis between RA and normal samples.**

**Fig 5. The co-expression network of signature genes**
